# Correction: ExoS effector in *Pseudomonas aeruginosa* Hyperactive Type III secretion system mutant promotes enhanced Plasma Membrane Rupture in Neutrophils

**DOI:** 10.1371/journal.ppat.1014353

**Published:** 2026-06-22

**Authors:** Arianna D. Reuven, Sarah Katzenell, Bethany W. Mwaura, James B. Bliska

In Fig 9C of [1], the Total H3, Cit H3, and β-actin panels are flipped along the horizontal axis. The corrected Fig 9 is provided with this notice, and the original blots are provided in [2].

**Fig 9:**
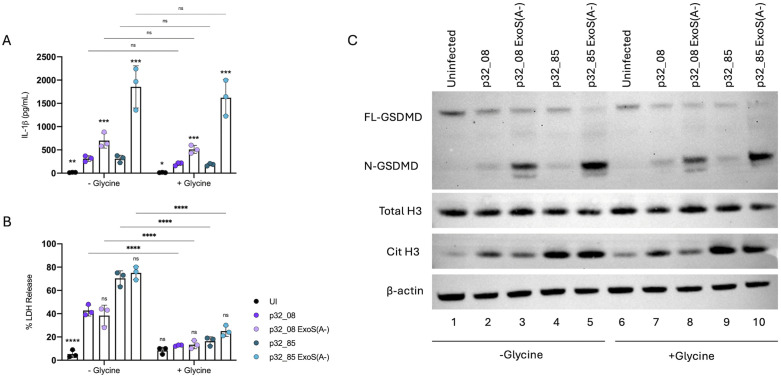
Analysis of B6 BMNs infections with patient 32 strains in absence or presence of glycine. B6 BMNs were left uninfected/UI or infected for 60 min with indicated strains at MOI 10 in the absence or presence of 5 mM glycine and analyzed for released IL-1β (A) or LDH **(B)**. Data represent normalized values for 2.5x10^5^ cells/well ± the standard deviation from three independent experiments. **(C)** Samples of total well contents were analyzed by immunoblotting for full length (FL-) or cleaved (N-) GSDMD, total histone 3 (H3), citrullinated histone 3 (Cit H3), and β-actin as a loading control. One representative blot of three independent experiments is shown. **(A, B)** Significant differences were determined by two-way ANOVA comparing p32_08 to UI or p32_08(ExsA-) or comparing p32_85 to p32_85 ExsA(A-) within groups or comparing between conditions as shown by brackets. ns, not significant; * P < 0.05; ** P < 0.01; *** P < 0.001; **** P < 0.0001.
