# Supplementary material for: ExoS effector in Pseudomonas aeruginosa Hyperactive Type III secretion system mutant promotes enhanced Plasma Membrane Rupture in Neutrophils
Source: PLoS Pathog. 2025 Apr 2;21(4):e1013021. doi: 10.1371/journal.ppat.1013021 (PMC11984736; doi:10.1371/journal.ppat.1013021)
Supplement: S1 Table — (PDF) [file ppat.1013021.s001.pdf]

Table S1: Primers

Primer Description	Primer Sequence
ExoS Forward with SacI site	GGgagctcCAACCATCATGCATATTCAATCGC
ExoS Reverse with EcoRI site	GGGAATTCTTCGTCATCCTCAATCCGTACG
ExoS(A-) mutagenesis Forward	GGATATCGAACTACAAGAATgacAAAgacATTCTCTATAACAAAGAAACC
ExoS(A-) mutagenesis Reverse	GGTTTCTTTGTTATAGAGAATgtcTTTgtcATTCTTGTAGTTCGATATCC
ExoS Sequencing	CAAGGCGCTGGCGGATGG
ExsA Forward with SacI site	GAGCTCGCATTTCGAGGGGCGTTCGG
ExsA Reverse with EcoRI site	GAATTCCCGCGCAGAGGAGAATCTG
ExsA^{T48I} Forward with XmaI site	CATCGATTCCATTTTTTGCCTGGCCCCGGGCGAGTTGC
ExsA^{T48I} Reverse with XmaI site	GCAACTCGCCCGGGGCCAGGCAAAAAATGGAATCGATG
ExsA Forward for BACTH	GCAGGGTCGACTCTAGAGATGCAAGGAGCCAAATCTC
ExsA Reverse for BACTH	CGAGCTCGGTACCCGGGGGTTATTTTTAGCCCGG
ExsD Forward for BACTH	TGCAGGTCGACTCTAGAGATGGAGCAGGAAGACGATAA
ExsD Reverse for BACTH	CGAGCTCGGTACCCGGGGGCTCTGCCAGTAGAAGTG
ExsA Sequencing	ATGCAAGGAGCCAAATCTCTTGG
M13 F	CAGGGTTTTCCCAGTCACGAC
M13 R	CACACAGGAAACAGCTATGAC
