# Supplementary material for: ExoS effector in Pseudomonas aeruginosa Hyperactive Type III secretion system mutant promotes enhanced Plasma Membrane Rupture in Neutrophils
Source: PLoS Pathog. 2025 Apr 2;21(4):e1013021. doi: 10.1371/journal.ppat.1013021 (PMC11984736; doi:10.1371/journal.ppat.1013021)
Supplement: S1 Appendix — (PDF) [file ppat.1013021.s002.pdf]

```
// 1. prompt the user to choose input and output folders, define the
file type, and define cell size and circularity
// 2. run an iterative functiuon that opens the input folder and looks
for the defined file type
//    if the file is the right type, run the "process file" function
// 3. the "process file" function calls on several functions in order:
//    3.1 CleanUp to clean up any previously open windows
//    3.2 OpenFile to open the file and turn it into a MAX projection
//        in the process taking note of the file name and the number
of channels
//    3.3 GetCellROIs - merges all channels, then sets a threshold to
create a master ROI of each cell no matter what color it is
//    3.4 ApplyCellROIs - splits the MAX projection into channels,
make it 8bit, apply the ROIs
//                 3.4.1 IsThisCellBright - ask what is the MFI of Ch1
and Ch2 in each ROI
//    3.5 save the MFI lists as an .xls
// 4. save the cell counts from the entire folder as a single .xls


// opens a window prompting the user to enter parameters

// define the input and output folders
#@ File (label = "Input directory", style = "directory") input_folder
#@ File (label = "Output directory: z_max", style = "directory") z_max
#@ File (label = "Output directory: merge", style = "directory") merge
#@ File (label = "Output directory: Mask", style = "directory") Mask
#@ File (label = "Output directory: CellCount", style = "directory")
CellCount
// define the file type to work on
#@ String (label = "File type", value = ".nd2") suffix

// define the cell parameters
#@ string (label = "lower threshold", value = "20") lower_threshold
#@ string (label = "cell size", value = "20-infinity") cell_size
#@ string (label = "cell circularity", value = "0.1-1.00")
cell_circularity

//-=-=-=-=-

//set the measurements you will use to analyze the particles
run("Set Measurements...", "area mean shape display add redirect=None
decimal=2");

ProcessFolder(input_folder, z_max, merge, Mask);

//-=-=-=-=-

// function to scan folders/subfolders/files to find files with
// the desired suffix and prepare a list of files to be processed
```

```
function ProcessFolder(input_folder, z_max, merge, Mask) {
  ListOfFiles = getFileList(input_folder);
  for (i = 0; i < ListOfFiles.length; i++) {
    file = ListOfFiles[i];

    if (File.isDirectory(input_folder + File.separator + file)) {
      ProcessFolder(input_folder + File.separator + file,
input_folder);
    }

    if (endsWith(file, suffix)) {
      save_data_name = ProcessFile(input_folder, z_max, merge, Mask,
file, cell_size, cell_circularity);
    }
  }
  // save the total cell counts from ALL IMAGES
  selectWindow("Summary");
  saveAs("Results", CellCount + File.separator +
"ALL_IMAGES_H_cell_counts.xls");
  run("Close");
}


// function to open a file, detect cells, make an ROI for each cell,
detect fluorescence intensity in the cell
// the function itterates over each file found in the folder
function ProcessFile(input_folder, z_max, merge, Mask, file,
cell_size, cell_circularity){

  // clean up any detritus from the previous file
  CleanUp();

  //open the next file, while also retrieving the image id and the
file name as variables
  array_image_identifiers = OpenFile(input_folder, file);
  image_id = array_image_identifiers[0];
  save_file_name = array_image_identifiers[1];
  save_data_name =
substring(array_image_identifiers[1],0,lengthOf(array_image_identifier
s[1])-2);

  // define a variable for the number of channels in the image
  selectWindow("MAX_" + save_file_name + suffix);
  n_channels = nSlices;

  // count the cells and generate an ROI around each cell
  n_cells = GetCellROIs(n_channels);

  // get the MFI of each channel inside each cell (ROI)
  ApplyCellROIs(n_channels);
```

```
  // save the MFI data for all the cells in this particular image
  // (the cell count for all images in the file will be save when the
entire folder has been processed)
  SaveData();

  // clean up any detritus from the previous file
  CleanUp();

  return;
}

//-=-=-=-=-=- below are all the mini-functions that are called


// clean up any previously generated results
function CleanUp(){
  // close all images
  close("*");
  // empty the ROI manager
  roiManager("reset");
  // empty the results table
  run("Clear Results");
  // close all windows. This does not close the log window, which
holds the cell count.
  run("Close All");
}

// open a file, take note of its name, and make a zstack
function OpenFile(input_folder, file){
        open(input_folder + File.separator + file);

    // take note of the file name (minus the suffix) and the file-ID
for the MAX project
        image_id=getImageID();
        file_name=getTitle();
        save_file_name=substring(file_name,0,lengthOf(file_name)-4);

        // make a max-projection of the z-stack
        run("Z Project...", "projection=[Max Intensity]");

        //select the original image, and close it
        selectWindow(file_name);
        close();

        return newArray(image_id, save_file_name);
}

// use the MAXproject image to generate ROIs around the cells, based
on all channels combined.
```

```
// it also saves images from each intermediate step for record keeping
and publication.
function GetCellROIs(n_channels){

        //select the appropriate image file
        selectWindow("MAX_" + save_file_name + suffix);

        // duplicate the image so you can make the ROIs without
messing up the original
        run("Duplicate...", "duplicate");

        // iterate the function over each channel, to adjust their
fluoresecence to a threshold
        // this allowes you to set a different saturation level for
overly dim/bright channels
        for (ch = 1; ch <= n_channels; ch++) {
                Stack.setChannel(ch);
                if (ch == 1) {
                   run("Enhance Contrast", "saturated=.35");
                }
                if (ch == 2) {
                        run("Enhance Contrast", "saturated=0.35");
                }
        }

        //save a z-projection with separate channels
        //saveAs("Tiff", z_max + File.separator + save_file_name +
"_A_zmax");

        // collapse the different colors into one layer
        Property.set("CompositeProjection", "Max");
    Stack.setDisplayMode("composite");
        run("Stack to RGB");
        //save a merged copy in color
        //saveAs("Tiff", merge + File.separator + save_file_name +
"_B_merge");

        // turn it into a BW 8-bit image
    run("8-bit");
        // save the BW 8bit intermediary image
        //saveAs("Tiff", Mask + File.separator + save_file_name +
"_C_BW8bit");

        //Adjust the threshold to say what counts as "real"
fluorescence and what counts as background
        setAutoThreshold("Default dark");
    run("Threshold...");
    setThreshold(lower_threshold, 255);

    //convert it to a mask so we can run analyze particles
```

```
    run("Convert to Mask");
    //saveAs("Tiff", CellCount + File.separator + save_file_name +
"_D_mask");

    //analyze the particles
    run("Analyze Particles...", "size=" + cell_size + " circularity="
+ cell_circularity + " show=[Overlay Masks] display summarize overlay
add composite");
    // get the number of cells from the ROI manager
    ROI_count = roiManager("count");

    //save the image showing the ROIs
   // Overlay.flatten
  //  saveAs("Tiff", CellCount + File.separator + save_file_name +
"_E_ROIs");

    // close the "masked" file
    close("MAX_" + save_file_name + "-1" + suffix);

    return(ROI_count)
}

// take the ROIs generated by GetROI and superimpose them on the raw,
unadjusted channels
// within it is another function, which asks what is the fluorescence
inside each ROI
function ApplyCellROIs(n_channels){

        //select the MAX projection from earlier
        selectWindow("MAX_" + save_file_name + suffix);
        //split the channels for easier processing
        run("Split Channels");

        // iterate the function over each channel, to adjust their
fluoresecence to a threshold
        for (ch = 1; ch <= n_channels; ch++) {

                //select the appropriate window
        selectWindow("C" + ch +"-MAX_" + save_file_name + suffix);

            // make it 8bit (this just gives the MFI in "easy"
numbers, but they will be arbitrary units either way)
                run("8-bit");
                //saveAs("Tiff", CellCount + File.separator +
save_file_name + "_F_mask-C" + ch);


                // apply the ROIs from the merged image
                run("From ROI Manager");
```

```
                // a function to measure the MFI of each channel in
each ROI
                IsThisCellBright(n_cells);

                //save a version with the ROIs drawn on it
                //selectWindow("C" + ch +"-MAX_" + save_file_name +
suffix);
                //Overlay.flatten
        //saveAs("Tiff", CellCount + File.separator + save_file_name +
"_G_ROI-C" + ch);


        }
}

// a function to measure the MFI of each channel in each ROI
function IsThisCellBright(n_cells){

    // iterate the function over each ROI, aka each cell
    for (c = 0; c < n_cells; c++) {
            roiManager("Select", c);
        // ask what is the MFI in the cell
        run("Measure");
    }
}

// save the MFI for each cell in this image
function SaveData(){
        selectWindow("Results");
        saveAs("Results", CellCount + File.separator + save_file_name
+ "_H_cell_MFI.xls");
        run("Close");
}
```
