# Supplementary material for: ExoS effector in Pseudomonas aeruginosa Hyperactive Type III secretion system mutant promotes enhanced Plasma Membrane Rupture in Neutrophils
Source: PLoS Pathog. 2025 Apr 2;21(4):e1013021. doi: 10.1371/journal.ppat.1013021 (PMC11984736; doi:10.1371/journal.ppat.1013021)
Supplement: S1 Fig — BMNs were isolated from B6 mice and analyzed immediately without staining (A), immediately with staining (B) or after 18 hr incubation without (C) or with (D) 100 ng/ml LPS. BMNs were stained with e780, Ly6G-PE and CD11b-FITC and analyzed by flow cytometry. (A-D) The gates indicate from left to right: singlets, normal size, live cells, and cells that are Ly6G+ and CD11b+. Histograms show overlay of fluorescence intensity for Ly6G+ (E) or CD11b+ (F) in each group. (PDF) [file ppat.1013021.s003.pdf]

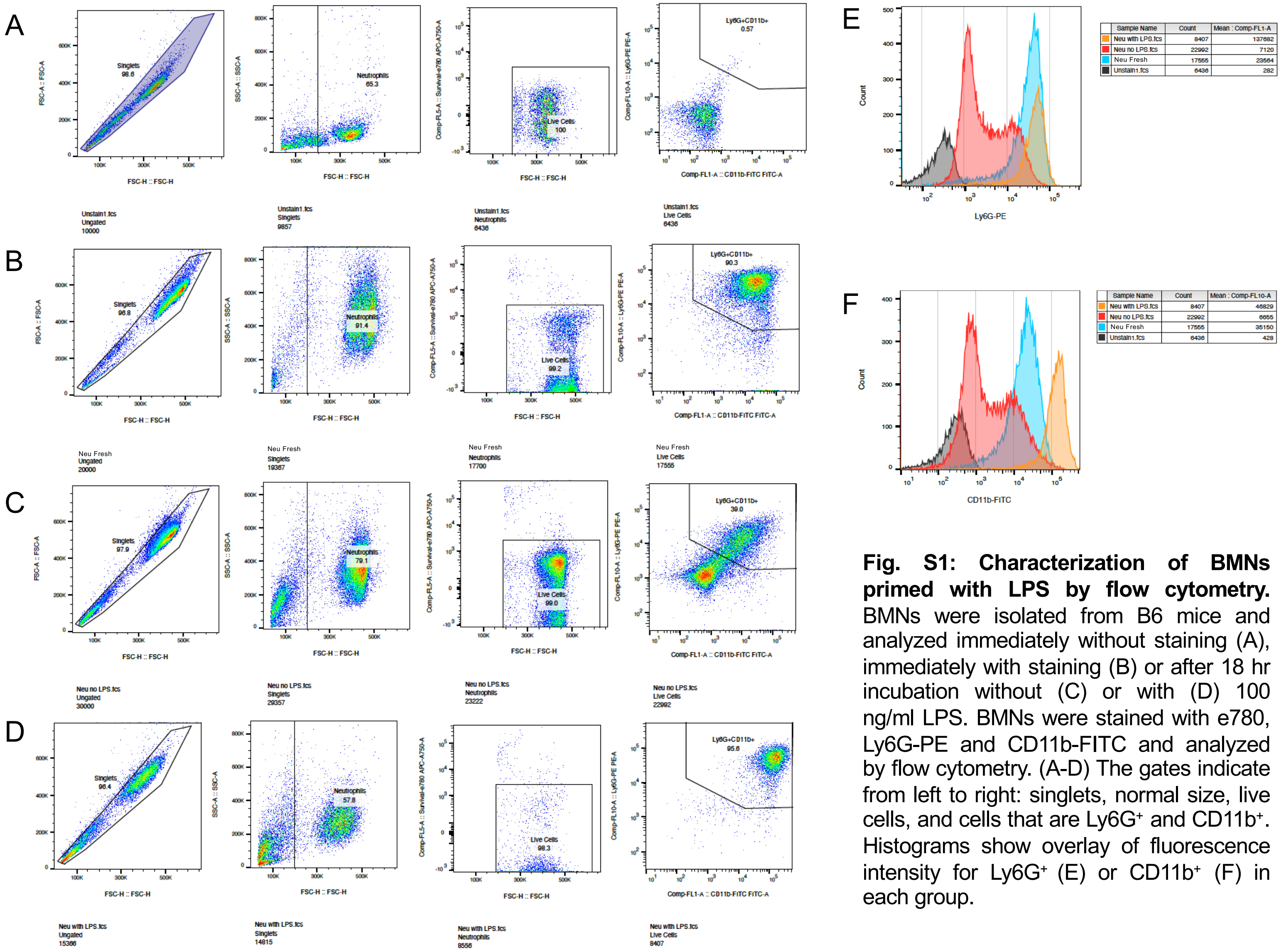

Fig. S1: Characterization of BMNs primed with LPS by flow cytometry. BMNs were isolated from B6 mice and analyzed immediately without staining (A), immediately with staining (B) or after 18 hr incubation without (C) or with (D) 100 ng/ml LPS. BMNs were stained with e780, Ly6G-PE and CD11b-FITC and analyzed by flow cytometry. (A-D) The gates indicate from left to right: singlets, normal size, live cells, and cells that are Ly6G$^+$ and CD11b$^+$. Histograms show overlay of fluorescence intensity for Ly6G$^+$ (E) or CD11b$^+$ (F) in each group.
