# Supplementary material for: ExoS effector in Pseudomonas aeruginosa Hyperactive Type III secretion system mutant promotes enhanced Plasma Membrane Rupture in Neutrophils
Source: PLoS Pathog. 2025 Apr 2;21(4):e1013021. doi: 10.1371/journal.ppat.1013021 (PMC11984736; doi:10.1371/journal.ppat.1013021)
Supplement: S2 Fig — B6 BMNs were left UI or infected for 60 min with PAO1F, ExoS(A-), ExoT(A-), or the ExoS(A-)ExoT(A-) double mutant at MOI 10 and analyzed for released IL-1β (A) or LDH (B). Data represent normalized values for 2.5x10^5^ cells/well ± the standard deviation from three independent experiments. Significant differences were determined by two-way ANOVA comparing to PAO1F. ns, not significant; * P<0.05; ** P<0.01. (PDF) [file ppat.1013021.s004.pdf]

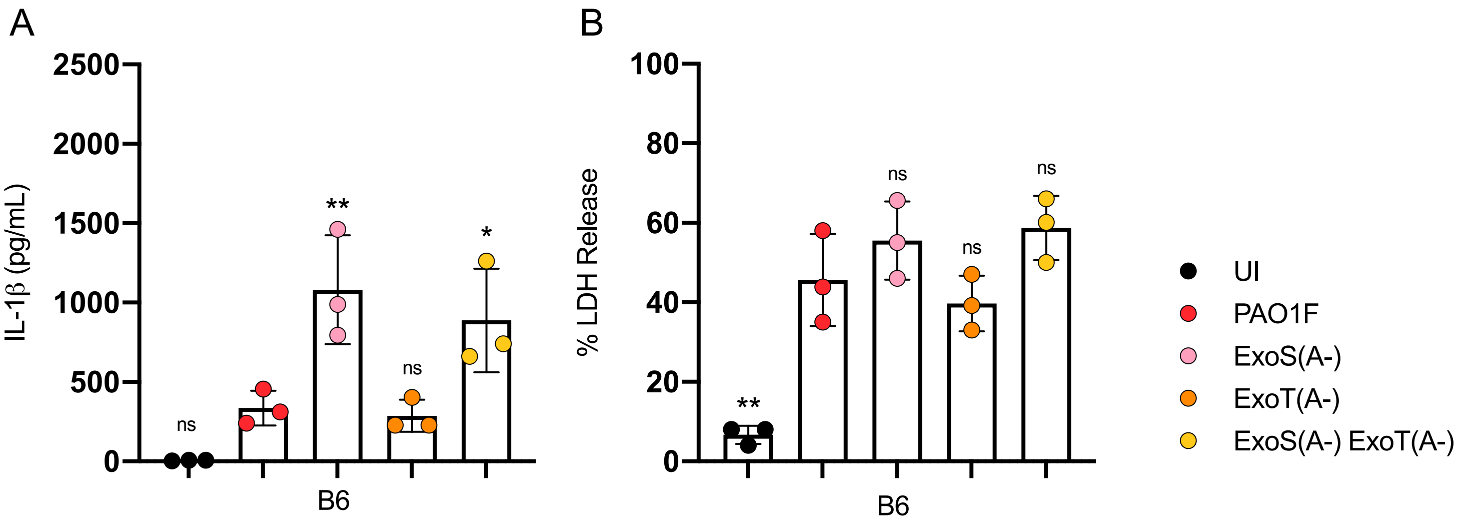

Fig. S2. Analysis of BMN infections with PAO1F or ExoS and/or ExoT ADPRT catalytic mutants. B6 BMNs were left UI or infected for 60 min with PAO1F, ExoS(A-), ExoT(A-), or the ExoS(A-)ExoT(A-) double mutant at MOI 10 and analyzed for released IL-1β (A) or LDH (B). Data represent normalized values for $2.5x10^5$ cells/well ± the standard deviation from four independent experiments. Significant differences were determined by two-way ANOVA comparing to PAO1F. ns, not significant; * P<0.05; ** P<0.01;.
