# Supplementary material for: ExoS effector in Pseudomonas aeruginosa Hyperactive Type III secretion system mutant promotes enhanced Plasma Membrane Rupture in Neutrophils
Source: PLoS Pathog. 2025 Apr 2;21(4):e1013021. doi: 10.1371/journal.ppat.1013021 (PMC11984736; doi:10.1371/journal.ppat.1013021)
Supplement: S3 Fig — The indicated strains of P. aeruginosa were grown in bacterial media that activates the T3SS. Secreted ExoS was collected from supernatants and detected by immunoblotting using anti-ExoS antibody. In (A) indicated strains of PAO1F and p32 isolates were analyzed. In (B) left lower ExsDWT and left upper ExsDT188P strains were analyzed. In (C) three independent p32_08 ExsAT48I and two independent p32_85 ExsAWT allele swap mutants were analyzed along with one parental control (WT or T48I) strain for each. (PDF) [file ppat.1013021.s005.pdf]

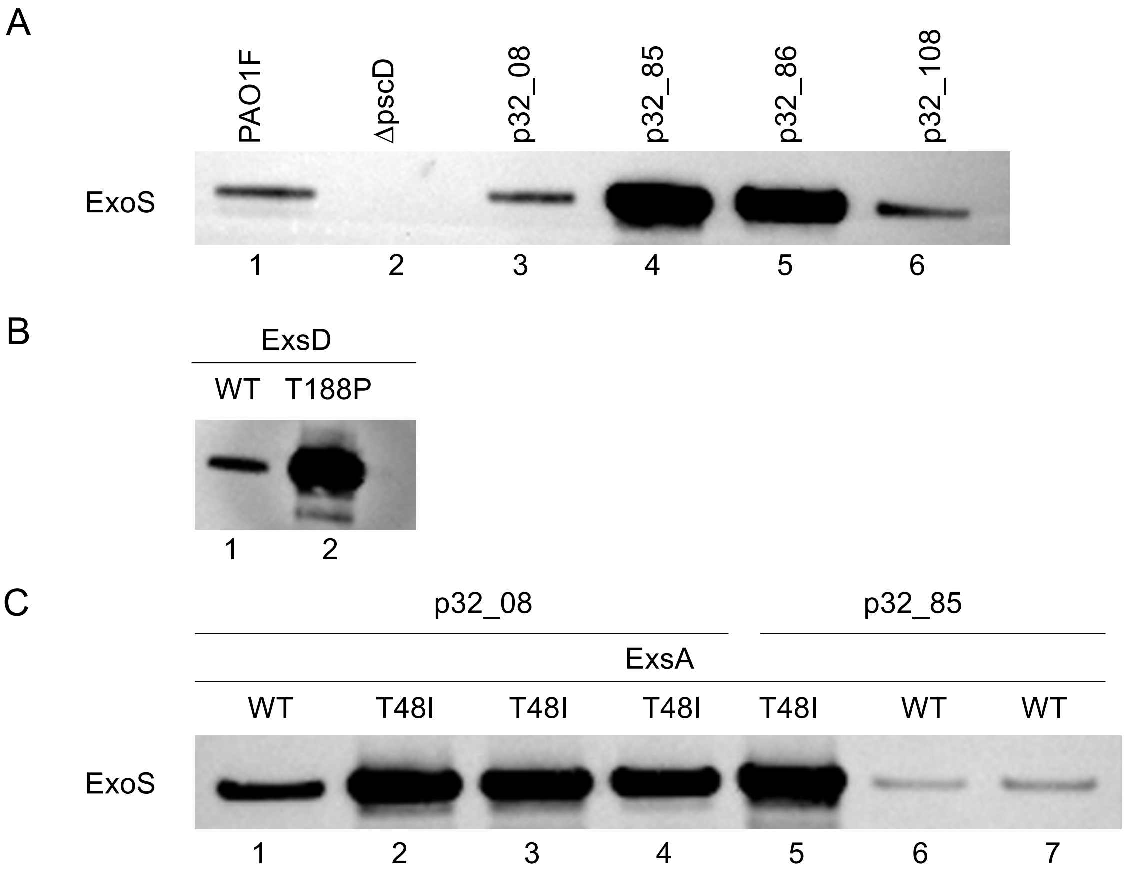

Fig. S3: Analysis of ExoS secreted by *P. aeruginosa* isolates. The indicated strains of *P. aeruginosa* were grown in bacterial media that activates the T3SS. Secreted ExoS was collected from supernatants and detected by immunoblotting using anti-ExoS antibody. In (A) indicated strains of PAO1F and p32 isolates were analyzed. In (B) left lower $ExsD^{WT}$ and left upper $ExsD^{T188P}$ strains were analyzed. In (C) three independent p32_08 $ExsA^{T48I}$ and two independent p32_85 $ExsA^{WT}$ allele swap mutants were analyzed along with one parental control (WT or T48I) strain for each.
