# Supplementary material for: ExoS effector in Pseudomonas aeruginosa Hyperactive Type III secretion system mutant promotes enhanced Plasma Membrane Rupture in Neutrophils
Source: PLoS Pathog. 2025 Apr 2;21(4):e1013021. doi: 10.1371/journal.ppat.1013021 (PMC11984736; doi:10.1371/journal.ppat.1013021)
Supplement: S4 Fig — B6 BMNs were left UI or infected for 60 min with laboratory strains PAO1F or its T3SS null mutant ∆pscD or p32 isolates p32_08, p32_85, p32_86, or p32_108 at MOI 10 (A, B) and analyzed for released IL-1β (A) or LDH (B). Data represent normalized values for 2.5x10^5^ cells/well ± the standard deviation from 3 independent experiments. Significant differences were determined by one-way ANOVA comparing to PAO1F for UI or ∆pscD, or comparing to p32_08 for other patient 32 isolates, or comparing between conditions as shown by brackets. ns, not significant; * P<0.05; ** P<0.01; *** P<0.001. (PDF) [file ppat.1013021.s006.pdf]

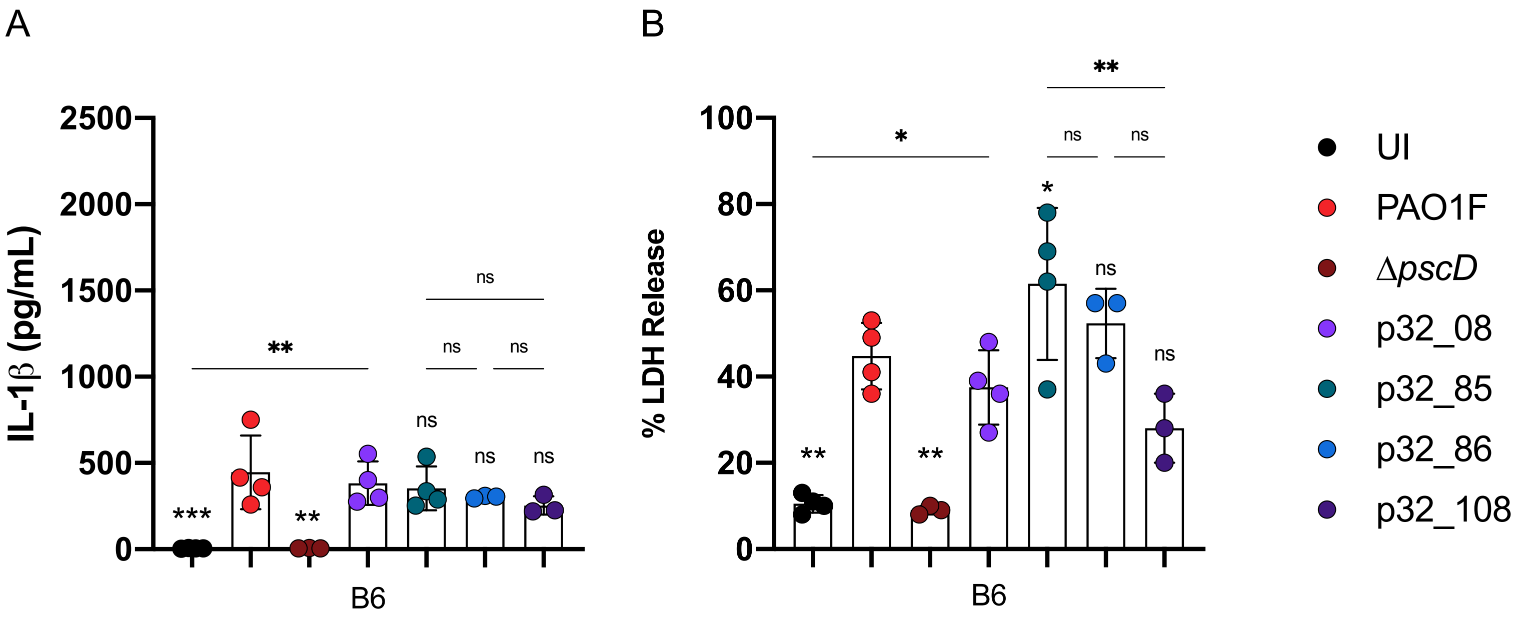

Fig. S4: Analysis of BMN infections with PAO1F strains or patient 32 isolates. B6 BMNs were left UI or infected for 60 min with laboratory strains PAO1F or its T3SS null mutant Δ*pscD* or p32 isolates p32_08, p32_85, p32_86, or p32_108 at MOI 10 (A, B) and analyzed for released IL-1β (A) or LDH (B). Data represent normalized values for 2.5×10^5 cells/well ± the standard deviation (A, B) 3 independent experiments (A, B). Significant differences were determined by one-way ANOVA comparing to PAO1F for UI or Δ*pscD,* or comparing to p32_08 for other patient 32 isolates, or comparing between conditions as shown by brackets. ns, not significant; * P<0.05; ** P<0.01; *** P<0.001.
