# Supplementary material for: ExoS effector in Pseudomonas aeruginosa Hyperactive Type III secretion system mutant promotes enhanced Plasma Membrane Rupture in Neutrophils
Source: PLoS Pathog. 2025 Apr 2;21(4):e1013021. doi: 10.1371/journal.ppat.1013021 (PMC11984736; doi:10.1371/journal.ppat.1013021)
Supplement: S6 Fig — (A) ExsA is shown in green with position of T48I. ExsD is shown in magenta with positions of T188P [24] and S164P [23]. (B) E. coli B2H reporter strains containing pairs of vectors encoding the indicated Exs or Zip proteins or empty vectors (EV) were assayed for production of β-galatosidase as measured in Miller Units. Each data point is from an independent experiment. Significant differences as compared to ExsA+ExsD was determined by one-way ANOVA. ns, not significant; **** P<0.0001. (PDF) [file ppat.1013021.s008.pdf]

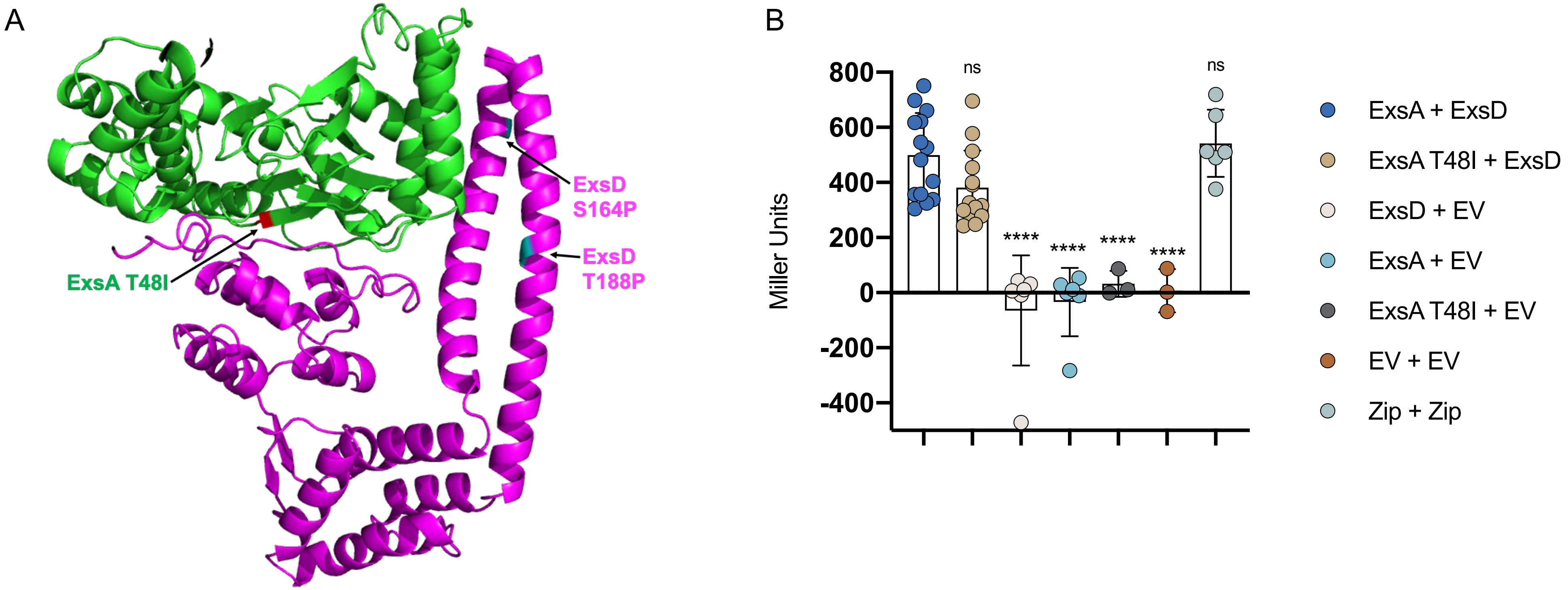

Fig. S6: AlphaFold2 prediction of ExsA-ExsD complex and bacterial two hybrid assay of ExsA-ExsD interaction. (A) ExsA is shown in green with position of T48I. ExsD is shown in magenta with positions of T188P (Jorth et al. 2015) and S164P (Jorth et al. 2021). (B) *E. coli* B2H reporter strains containing pairs of vectors encoding the indicated Exs or Zip proteins or empty vectors (EV) were assayed for production of β-galatosidase as measured in Miller Units. Each data point is from an independent experiment. Significant differences as compared to ExsA+ExsD was determined by one-way ANOVA. ns, not significant; **** P<0.0001.
