# Supplementary material for: ExoS effector in Pseudomonas aeruginosa Hyperactive Type III secretion system mutant promotes enhanced Plasma Membrane Rupture in Neutrophils
Source: PLoS Pathog. 2025 Apr 2;21(4):e1013021. doi: 10.1371/journal.ppat.1013021 (PMC11984736; doi:10.1371/journal.ppat.1013021)
Supplement: S7 Fig — (A) Hyperactive T3SS ExoS+ P. aeruginosa translocates flagellin and active ExoS into neutrophil. ExoS inhibits phagocytic signaling by targeting host proteins such as Ras. ExoS inhibits assembly of NLRC4 inflammasome in response to flagellin detection by NAIP. ExoS promotes formation of unknown pores in plasma membrane, allowing influx of Ca2+. Ca2+ enters the nucleus to activate Pad4, resulting in Cit3 generation, chromatin decondensation into cytosol, and incomplete NET extrusion due to maintenance of the cortical actin cytoskeleton. Pore formation triggers NINJ1 activation, PMR and release of LDH and HMGB1. Glycine inhibits activation of NINJ1 to prevent PMR. (B) Hyperactive T3SS ExoS ADPRT- P. aeruginosa translocates flagellin and inactive ExoS into neutrophil. ExoS ADPRT- fails to inhibit phagocytic signaling and assembly of NLRC4 inflammasome. ExoS ADPRT- fails to promote pore formation and unleashes caspase-1 to cleave pro-IL-1β and GSDMD. N-GSDMD forms plasma membrane pores, allowing Ca2+ to enter the nucleus to activate Pad4, resulting in Cit3 generation, chromatin decondensation into cytosol, and incomplete NET extrusion. GSDMD pore formation triggers NINJ1 activation, PMR and release of LDH and HMGB1. Glycine inhibits activation of NINJ1 to prevent PMR. (PDF) [file ppat.1013021.s009.pdf]

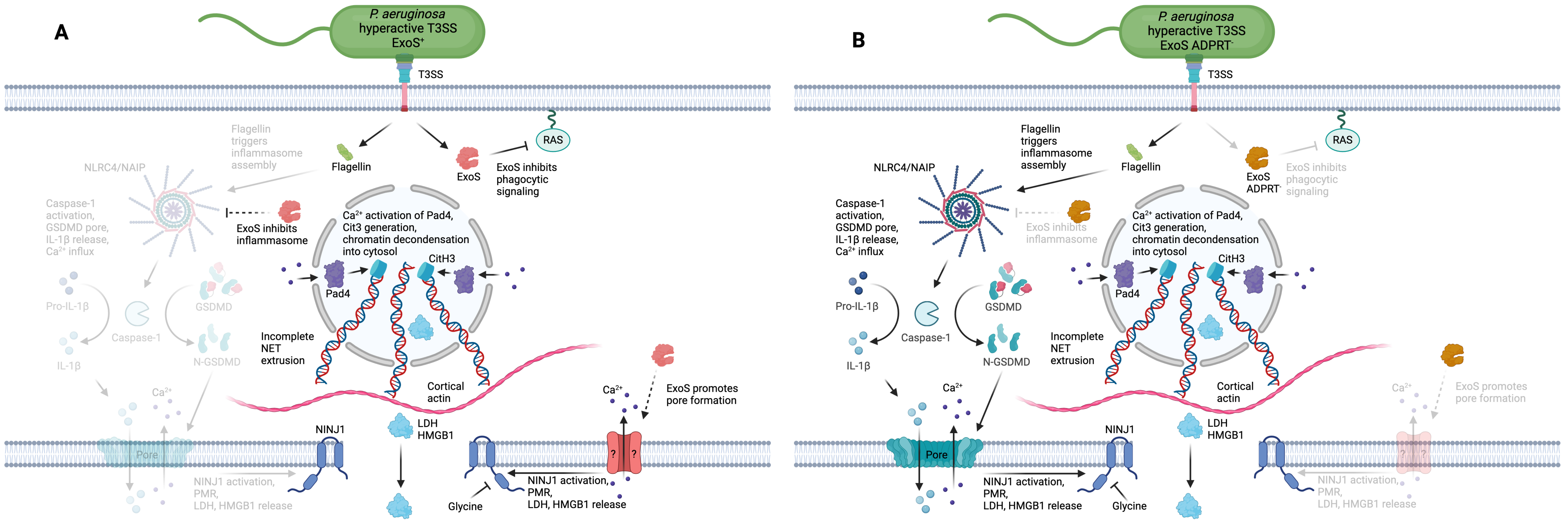

Fig. S7: Model of neutrophil responses to hyperactive T3SS ExoS$^+$ or ExoS ADPRT$^-$ *P. aeruginosa.* (A) Hyperactive T3SS ExoS$^+$ *P. aeruginosa* translocates flagellin and active ExoS into neutrophil. ExoS inhibits phagocytic signaling by targeting host proteins such as Ras. ExoS inhibits assembly of NLRC4 inflammasome in response to flagellin detection by NAIP. ExoS promotes formation of unknown pores in plasma membrane, allowing influx of Ca^{2+}. Ca^{2+} enters the nucleus to activate Pad4, resulting in Cit3 generation, chromatin decondensation into cytosol, and incomplete NET extrusion due to maintenance of the cortical actin cytoskeleton. Pore formation triggers NINJ1 activation, PMR and release of LDH and HMGB1. Glycine inhibits activation of NINJ1 to prevent PMR. (B) Hyperactive T3SS ExoS ADPRT$^-$ *P. aeruginosa* translocates flagellin and inactive ExoS into neutrophil. ExoS ADPRT$^-$ fails to inhibit phagocytic signaling and assembly of NLRC4 inflammasome. ExoS ADPRT$^-$ fails to promote pore formation and unleashes caspase-1 to cleave pro-IL-1β and GSDMD. N-GSDMD forms plasma membrane pores, allowing Ca^{2+} to enter the nucleus to activate Pad4, resulting in Cit3 generation, chromatin decondensation into cytosol, and incomplete NET extrusion. GSDMD pore formation triggers NINJ1 activation, PMR and release of LDH and HMGB1. Glycine inhibits activation of NINJ1 to prevent PMR.
