# Supplementary material for: ExoS effector in Pseudomonas aeruginosa Hyperactive Type III secretion system mutant promotes enhanced Plasma Membrane Rupture in Neutrophils
Source: PLoS Pathog. 2025 Apr 2;21(4):e1013021. doi: 10.1371/journal.ppat.1013021 (PMC11984736; doi:10.1371/journal.ppat.1013021)
Supplement: Attachment — Submitted filename: Reuven PLoS 2024 Responses v3.docx [file ppat.1013021.s010.docx]

We thank the Reviewers and Editors for positive and constructive feedback. We have revised the manuscript in response to the comments as detailed below.

**Additional Editor Comments:**

The reviewers have found that the evidence largely support the title of the manuscript. However, reviewers #2 and 3 found that there are possible issues with the clinical strains that go beyond T3SS. In addition to addressing their specific comments, the addition of a mutant lacking T3SS would greatly support the effect is solely due to T3SS and ExoS.

**Response:** We have added new data that helps establish that, other than the codon change in *exsA* resulting in a hyperactive T3SS phenotype, there are no other relevant genetic differences between the clinical isolates. Most importantly, new Fig.S5 has neutrophil infection data with allele swap variants of the clinical isolates and shows that the *exsA* T48I codon change is required for enhanced plasma membrane rupture.

**Reviewers' Comments:**

Reviewer's Responses to Questions

**Part I - Summary**
Please use this section to discuss strengths/weaknesses of study, novelty/significance, general execution and scholarship.

Reviewer #1: The manuscript by Reuven et al. carefully examines the interaction of P. aeruginosa strains producing they type III secreted effector ExoS with neutrophils. Importantly for researchers in the cystic fibrosis arena, they both examine the interaction with bone-marrow derived neutrophils from wild type and deltaF508 mice. They also examine the effect of an exsD mutant that results in up-regulation of the T3SS and was identified in a CF patient isolate, further increasing the relevance for this patient group. The paper slots in nicely with a few recent reports, all cited in the manuscript, that analyze inflammasome activation in neutrophils infected by PAO1. The authors do a more careful analysis of the chromatin decondensation/induction of NETosis, and also expand on the role of ninjurin1 in plasma membrane rupture that occurs upon infection. In the end, the authors present evidence for three processes: 1) GSDM-dependent, ExoS-inhibited, NINJ1-independent IL1-beta release, 2) ExoS-ADPR-independent, but secretion-activity regulated, NINJ1-dependent PMR, and 3) ExoS-ADPR and NINJ1 independent, but secretion-activity regulated H3 citrulination. This, in conjunction with work by other labs that infer a role for NLRP3 in some of these processes begins to show the complicated nature of the interaction of P. aeruginosa with this key immune cell and helps focus future research trying to unravel these related processes and hopefully put them in the context of PA pathogenesis. The experiments were generally well-presented and explained.

Reviewer #2: The manuscript explores the role of ExoS of hyperactive T3SS Pseudomonas aeruginosa mutants in promoting neutrophil plasma membrane rupture and incomplete neutrophil extracellular trap (NET) extrusion via NLRC4-independent manner. The study utilizes bone marrow derived murine neutrophils along with clinical isolates obtained from people with cystic fibrosis (CF) and laboratory strain PAO1F. Using LPS-primed neutrophils, the authors have demonstrated that ADPRT activity of ExoS suppresses IL-1β release by neutrophils challenged with PAO1F. The authors have also identified a CF isolate that is a hyperactive T3SS strain due to a mutation in exsA, a transcription factor of ExoS. The hyperactive T3SS mutant promotes citrullination of histone H3, nuclear decondensation, incomplete NET extrusion and plasma membrane rupture. The authors have further demonstrated that plasma membrane rupture occurs independent of caspase-1 activation as well as inflammasome adaptor ASC and instead is a result ninjurin. Overall, the manuscript is well written and addressing an important topic. There are some issues raised below that should be considered prior to publication.

Reviewer #3: The interaction of Pseudomonas aeruginosa with neutrophils is an important topic of investigation allowing us to further understand the pathogenesis of infection and pathology in the airways and other infection sites. The authors used a hyperactive type III secretion mutant isolated from a CF patient to investigate the effects of ExoS ADPR activity on inflammasome activation and plasma membrane rupture (PMR) in murine neutrophils with and without inflammasome components (mutants in caspase 1 or ASC) and compared wild-type with engineered CF neutrophils with delta F508 mutation. Results appeared to be confirmatory of multiple prior studies of ExoS effects on neutrophil function and inflammasome activity but showed that ExoS effects on PMR (cytotoxicity LDH release) were relatively independent from inflammasome (IL-1beta) changes throughout the study. PMR was inhibited by glycine suggesting involvement of injurin-1 in PMR.

The strengths of the study are the genetic analysis of the mechanism for T3SS hyper-secretion in the CF mutant, the consistent delineation of bacterial ExoS mediated PMR from effects on inflammasome modulation throughout the study, and the likely mechanism for PMR involving ninjurin-1. The manuscript was well written and thoughtfully discussed. Strong execution and scholarship consistent with this excellent research group.

Weaknesses per se could be the reliance on two clonal isolates from one patient, the lack of a defined exsD mutant control, unclear as to bacterial location (intracellular vs. extracellular) when interacting with neutrophils and absence of imaging quantification in Figs. 5 and 6. Also, it appeared that some of the activity of ExoS attributed to ADPr activity was similar with ExoS ADFPr mutant, e.g. Fig. 8B.

**Response:** We have addressed each of these concerns in detail below.

**Part II – Major Issues: Key Experiments Required for Acceptance**
Please use this section to detail the key new experiments or modifications of existing experiments that should be absolutely required to validate study conclusions.

Generally, there should be no more than 3 such required experiments or major modifications for a "Major Revision" recommendation. If more than 3 experiments are necessary to validate the study conclusions, then you are encouraged to recommend "Reject".

Reviewer #1: n/a

Reviewer #2:

L141-143, L307-309: The authors claim that the ADPRT activity of ExoS is necessary for PAO1 to inhibit NLRC4 inflammasome activation and IL-1β release. While the data in Fig. 2A demonstrates the latter, there is no direct evidence in Fig. 2 that demonstrates inhibition of NLRC4 inflammasome activation. It would seem important to include data demonstrating that NLRC4 is no longer inhibited by a catalytically inactive ExoS mutant to support the authors’ claim. There should also be some discussion regarding the PMR data in Fig. S2B with respect to the ExoT mutant effect. Was this expected?

**Response:** We agree there is no direct evidence that ExoS ADPRT activity is inhibiting the NLRC4 inflammasome. We are extrapolating from data in: 1) Minns et al. (Ref. 16) showing that NLRC4 is required for caspase-1 cleavage, IL-1β release, and LDH release in neutrophils infected with a PAO1 strain missing ExoS; and 2) our Fig.8C showing that ExoS ExoS ADPRT activity is required to inhibit caspase-1 cleavage in neutrophils infected with *P. aeruginosa*. In theory one could determine if ExoS ADPRT activity inhibits assembly of NLRC4 inflammasomes by fluorescence microscopy, but this could be technically challenging in neutrophils. We have added a sentence to discuss that the PMR data with the ExoT mutant did not trend differently from PAO1 (Fig.S2B). This was the expected result since Minns et al. (Ref. 16) showed that neutrophils infected with an ExoT ADPRT mutant do not release more IL-1β as compared to PAO1 and we obtained the same result (Fig.S2A).

L148. The results in Fig. 2 indicate that the CFTR genotype does not appear to be involved in IL-1β release from neutrophils. The rationale in the next section was to increase the biological significance of the results so additional CF isolates were investigated. These seem at odds with each other.

**Response:** The results in Fig.2 were obtained with PAO1 strains only but the point is well taken. We have revised the rationale at the beginning of the next section to “To determine if the use of clinical isolates would impact our results…”.

L176-183. Granted this likely took some time to accomplish these experiments but I’m not certain this adds anything additional to the main message of the paper. What experiments proposed in lines 181-183 would be conducted to get at this question?

**Response:** We think the results of these B2H experiments are important to include for completeness. We have added a sentence to the relevant section of the Discussion indicating that in vitro transcription assays (Ref. 32) could be used to be used to determine if the T48I codon change in ExsA relieves negative regulation by ExsD.

L185/196. Have the genome sequences of these hyperactive mutants been determined? Are the differences between these strains only due to the T48I mutation in exsA? This is partially addressed in Fig. S3B with cross complementation, but these studies evaluated ExoS levels. Might there be alterations outside exsA that could account for the data in Fig. 3/4?

**Response:** Yes, the genomic sequences of the clinical isolates used in our study were determined (Ref. 29) and we now mention this when the strains are introduced. In Ref. 29 only virulence genes with non-synonymous mutations within coding regions that were different between isolates were reported. As compared to the control strain p32_08 the only reported changes in the hyperactive mutant p32_85 are the T48I mutation in *exsA* and a V190L codon change in the gene *aaaA,* which encodes an arginine-specific autotransporter. To determine if the T48I mutation in *exsA* in the hyperactive mutant p32_85 is sufficient for the enhanced PMR data in Fig.3 we carried out neutrophil infections with *exsA* allele swap strains used for analysis of ExoS secretion (Fig. S3C) and describe this data in the manuscript. As shown in new Fig.S5B, changing *exsA*^T48I^ to the wildtype (WT) allele in p32_85 reversed the enhanced PMR phenotype in BMNs. These results indicate that the T48I codon change in p32_85 is sufficient to cause enhanced PMR in infected BMNs.

L207-209: The authors state that citH3 levels were elevated in neutrophils infected with p32_85 (hyperactive T3SS) compared to p32_08. The result is puzzling as neutrophils infected with the catalytic mutant ExoS(A-) also exhibits increased citH3. It is likely that the clinical isolate p32_85 may harbor additional pathoadaptive mutations in addition to that in exsA. Related to the above point, can the authors comment on whether p32_85 is genetically identical to PAO1F with the exception of exsA? Alternatively, constructing a hyperactive T3SS mutant in PAO1F background and challenging the neutrophils to examine whether increased citH3 levels are indeed a result of the ExoS ADPRT activity in the hyperactive T3SS mutant and is independent of NLRC4 activation.

**Response:** We agree that it can be puzzling that the hyperactive T3SS mutant p32_85 and an ExoS ADPRT catalytic mutant both increase CitH3 levels. As indicated in the manuscript we hypothesize that two distinct pathways can promote enhanced CitH3. In neutrophils infected with the hyperactive T3SS mutant p32_85 ExoS ADPRT activity promotes enhanced CitH3 while simultaneously inhibiting the NLRC4 pathway. In neutrophils infected with an ExoS ADPRT catalytic mutant the flagellin-NLRC4 pathway is unleashed to cause enhanced CitH3. We have created a model figure to illustrate these two distinct mechanisms resulting in the same outcome (Fig.S7). See response to above question concerning the question about p32_85 having additional pathoadaptive mutations. We have not determined the genetic conservation between p32_85 and PAO1F. We agree that constructing a hyperactive T3SS mutant in the PAO1F background would be informative, but we feel it may be unnecessary given the results of our allele swap data from neutrophil infections (Fig.S5) as discussed in the response to the question above.

Fig.4. Quantification of immunoblot depicting levels of H3 and citH3 is required as the H3 signal for ExoS(A-) lane is more intense than the other H3 signals. This makes it difficult to interpret the higher signal intensity of citH3 for ExoS(A-) lane. Quantification of citH3 signal normalized to H3 signal is recommended. This analysis can be extended to other components of the blot to strengthen the observations.

**Response:** We agree and have added to Fig.4 signal intensity values for CitH3 normalized to H3 from three independent experiments.

Fig.6. Quantification of the microscopy images depicting chromatin decondensation and NET extrusion is required to support the statement in L213-214 that hyperactive T3SS mutant P. aeruginosa promotes nuclear decondensation and incomplete NET extrusion in BMNs. It is difficult to distinguish if there are differences in these events between a ExoS (A-) strain and a hyperactive T3SS strain.

**Response:** We agree and have added quantification for chromatin decondensation and NET extrusion in Fig.5 and Fig.6 and revised the discussion of this data. Fig.6A-E shows that PAO1F ExoS(A-) and hyperactive T3SS strain p32_85 have similar phenotypes for chromatin decondensation and incomplete NET extrusion in BMNs. These results support our conclusion that an ExoS(A-) strain and a hyperactive T3SS strain both primarily cause chromatin decondensation and incomplete NET extrusion in BMNs, but by two distinct mechanisms, flagellin-NLRC4-caspase-1, and ExoS ADPRT, respectively (Fig.S7).

L253-254: The authors conclude from Figure 7 that the ExoS ADPRT is promoting PMR using LDH assay. In Fig. 8B, when using the hyperactive T3SS strain p32_85 and its corresponding ExoS (A-) mutant, the LDH released by neutrophils challenged by both strains is comparable, suggesting that ExoS ADPRT is not involved in PMR when looking at this hyperactive T3SS strain.

**Response:** In Fig.8B it is true that the hyperactive T3SS strain p32_85 and its corresponding ExoS (A-) mutant cause comparable LDH released in B6 BMNs. However as shown in Fig.8B in Casp1^-/-^ BMNs the hyperactive T3SS strain p32_85 continues to cause high LDH release while this phenotype is significantly reduced with the ExoS (A-) mutant. These results support our conclusion that ExoS ADPRT activity is promoting PMR independent of caspase-1 (Fig.S7A).

L264-265: Although it is reported that ExoS ADPRT activity is necessary to inhibit NLRC4 activation, the figure corresponding to this data (Fig. 8A, C) shows increased IL-1β release and cleavage of Gasdermin D, both of which are not exclusive to NLRC4 activation. Direct evidence using an anti-NLRC4 for immunoblot would seem to be required to support the statement.

**Response:** Fig.8A,C shows increased IL-1β release and cleavage of Gasdermin D with ExoS (A-) mutants, consistent with ExoS ADPRT activity inhibiting NLRC4 activation. We agree there is no direct evidence that ExoS ADPRT activity inhibits NLRC4 activation, and we are extrapolating based on available evidence including caspase-1 cleavage (Fig.8C) (see response to the first comment of Reviewer 2 above**)**. To our knowledge the activation status of NLRC4 is not routinely measured by immunoblotting.

Fig 8 and 9. PAO1F and ExoS (A-) control strains seem to be missing from the experiments. Since the experiments for these figures use clinical isolates that are resulting in unexpected results as stated by the authors in L269-271, it would seem pertinent to use laboratory WT strain and its mutant to validate these observations. Furthermore, in L269-271, the authors compare the observations of laboratory parent strain PAO1F and its ExoS (A-) mutant in previous figures (Fig.4 and 7) of the manuscript which further demonstrates the need for including these control strains in the experiments for Figures 8 and 9 for comparison.

**Response:** We understand the point that it may have been informative to include the laboratory parent strain PAO1F and its ExoS (A-) mutant in Fig.8 and 9 for comparison. Instead of repeating the experiments in Fig.8 and 9 with PAO1F and its ExoS(A-) we have added two new supplemental figures with BMN infection data (Fig.S4, S5) which help validate phenotypes with the clinical isolates.

Reviewer #3:

1. Were the clonal isolates from patient 32 examined for defects or hyperactivity in any other virulence mechanisms/factors? Was there only T3SS hyper-secretion?

**Response:** The genomic sequences of the clinical isolates used in our study were determined (Ref. 29) and we now mention this when the strains are introduced. In Ref. 29 only virulence genes with non-synonymous mutations within coding regions that were different between isolates were reported. As compared to the control strain p32_08 the only reported changes in the hyperactive mutant p32_85 are the T48I mutation in *exsA* and a V190L codon change in the gene *aaaA,* which encodes an arginine-specific autotransporter. The patient 32 isolates have not been examined for defects or hyperactivity in virulence mechanisms/factors other than T3SS activity and survival during BMN infection, but we plan to do this in the future.

2. The imaging in Figs. 5 and 6 could be quantified with statistical analysis for clearer representation of the findings?

**Response:** We agree and have added quantification for chromatin decondensation and NET extrusion in Fig.5 and Fig.6 and revised the discussion of this data. Fig.6A-E shows that PAO1F ExoS(A-) and hyperactive T3SS strain p32_85 have similar for phenotypes for chromatin decondensation and incomplete NET extrusion in BMNs. These results support our conclusion that an ExoS(A-) strain and a hyperactive T3SS strain both primarily cause chromatin decondensation and incomplete NET extrusion in BMNs, but by two distinct mechanisms, flagellin-NLRC4-caspase-1, and ExoS ADPRT, respectively (see new Fig.S7).

3. Was the bacterial location determined after neutrophil interaction? It was unclear if they were inside or outside of cells? A number of comments suggested they were internalized as might be expected.

**Response:** We have not determined the bacterial location after BMN interaction, but this is a high priority for future experiments.

4. Was a defined exsD mutant included at any time for comparison with the T3SS hyper-secretion clonal mutant?

**Response:** As shown in new Fig.S3B we have added ExoS hypersecretion data with an ExsD T188P mutant from Jorth et al. Ref. 24.

5. In Fig. 8B, the PMR (LDH) appeared similar in clonal isolate P32-85 and its ExoS ADPr mutant. This seems to contradict statement that ExoS ADPr activity is driving the process.

**Response:** In Fig.8B it is true that the hyperactive T3SS strain p32_85 and its corresponding ExoS (A-) mutant cause comparable LDH released in B6 BMNs. However as shown in Fig.8B in Casp1^-/-^ BMNs the hyperactive T3SS strain p32_85 continues to cause high LDH release while this phenotype is significantly reduced with the ExoS (A-) mutant. These results support our conclusion that ExoS ADPRT activity is promoting PMR independent of caspase-1 (Fig.S7A).

**Part III – Minor Issues: Editorial and Data Presentation Modifications**
Please use this section for editorial suggestions as well as relatively minor modifications of existing data that would enhance clarity.

Reviewer #1: Line 209 – could this be due to ExoS-dependent activation of NLRP3 (which seems to occur concomitantly with NLRC4 inactivation in neutrophils)?

**Response:** This seems unlikely under our infection conditions since Fig.4 shows that elevated CitH3 generation by the T3SS hyperactive mutant p32_85 is not associated with enhanced GSDMD cleavage as would be expected if NLRP3 was being activated concomitantly with NLRC4 inhibition.

Line 232 – sorry for being the non-immunologist idiot here. While I can see the puncta, I’m not sure I understand the explanation here. How does relaxed chromatin that is confined with in the BMN (small, bright foci) differ from decondensation without NET extrusion (cell-filling DNA stain)?

**Response:** We agree the explanation was not well described and to avoid confusion we have deleted this statement.

Fig. 7B. How come LDH release is up in the p32_85-infected cells that hypersecrete ExoS, when ExoS seems to interfere with LDH release in the case of the PAO1F strain?

**Response:** We agree that it can be puzzling that the hyperactive T3SS mutant p32_85 promotes LDH release (PMR) when ExoS ADPRT catalytic activity is proposed to inhibit PMR via the flagellin-NLRC4 pathway. As indicated in the manuscript we hypothesize that two distinct pathways can promote PMR. In neutrophils infected with the hyperactive T3SS mutant p32_85 ExoS ADPRT activity promotes PMR while simultaneously inhibiting the NLRC4 pathway. In neutrophils infected with an ExoS ADPRT catalytic mutant the flagellin-NLRC4 pathway is unleashed to cause PMR. We have created a model figure to illustrate these two distinct mechanisms resulting in the same outcome (Fig.S7).

Fig. 9. How specific is the glycine-mediated inhibition for Ninjurin-mediated plasma membrane rupture? The authors propose doing the experiments with ninjurin null cells as a follow-up experiment, which is reasonable, but I wonder if there is any information on this in the literature that can be cited to add perspective?

**Response:** One of the references we cite (Ref. 26) presents data that glycine does not protect against PMR from necroptosis, suggesting that the cytolytic activity of MLKL pores is not inhibited by glycine and providing some evidence for specificity.

297 (and 379?) - Could membrane-perturbation by translocon pore insertion combined with a ExoS-mediated inhibition of membrane damage repair be the trigger for NINJ1-dependent cell lysis? Control of H3 citrulination, however, would have to be controlled in a NINJ1 independent manner, perhaps by influx of calcium via the translocon. This may also relate to greater translocon insertion in the cells infected with the hyperactive mutant.

**Response:** It is an interesting hypothesis that the translocon pore could be important for ExoS-driven NINJ1-dependent PMR and calcium influx leading to CitH3. We have added a sentence to the Discussion to introduce this possibility. We also added a citation and discussion of a paper by Joe Barbieri (Ref. 39) which showed that ectopic expression of the active ADPRT domain of ExoS in cultured cells is sufficient to cause plasma membrane damage. We favor a model that ExoS promotes opening of a neutrophil channel to cause pore formation (Fig.S7A).

Reviewer #2:

Suggest replacing “CF patients” to individuals/people with CF wherever applicable (L35, 86, 150, 317). CFF is requesting this in publications and presentations.

**Response:** Done.

Fig 5 and 6: Suggest including scale bars in figure legend as the text is too small to read in the microscopy images.

**Response:** Done.

Reviewer #3:

The abstract could be clearer in representing the findings unique to the current study. Much of the abstract was dedicated to describing background studies rather than novel results.

**Response:** We understand the point and have complied with PLoS requirements to write an Author Summary after the abstract. The Author Summary is clearer in representing the novel and unique findings of the current study.
